# MicroRNAs in Hearing Disorders: Their Regulation by Oxidative Stress, Inflammation and Antioxidants

**DOI:** 10.3389/fncel.2017.00276

**Published:** 2017-09-11

**Authors:** Kedar N. Prasad, Stephen C. Bondy

**Affiliations:** ^1^Engage Global San Rafael, CA, United States; ^2^Center for Occupational and Environmental Health, Department of Medicine, University of California, Irvine Irvine, CA, United States

**Keywords:** MicroRNAs, hearing disorders, oxidative stress, inflammation, antioxidants

## Abstract

MicroRNAs (miRs) are small non-coding single-stranded RNAs that bind to their complimentary sequences in the 3′-untranslated regions (3′-UTRs) of the target mRNAs that prevent their translation into the corresponding proteins. Since miRs are strongly expressed in cells of inner ear and play a role in regulating their differentiation, survival and function, alterations in their expression may be involved in the pathogenesis of hearing disorders. Although increased oxidative stress and inflammation are involved in initiation and progression of hearing disorders, it is unknown whether the mechanisms of damage produced by these biochemical events on inner ear cells are mediated by altering the expression of miRs. In neurons and non-neuronal cells, reactive oxygen species (ROS) and pro-inflammatory cytokines mediate their damaging effects by altering the expression of miRs. Preliminary data indicate that a similar mechanism of damage on hair cells produced by oxidative stress may exist in this disease. Antioxidants protect against hearing disorders induced by ototoxic agents or adverse health conditions; however, it is unknown whether the protective effects of antioxidants in hearing disorders are mediated by changing the expression of miRs. Antioxidants protect mammalian cells against oxidative damage by changing the expression of miRs. Therefore, it is proposed that a similar mechanism of protection by antioxidants against stress may be found in hearing disorders. This review article discusses novel concepts: (a) alterations in the expression of miRs may be involved in the pathogenesis of hearing disorders; (b) presents evidence from neurons and glia cells to show that oxidative stress and pro-inflammatory cytokines mediate their damaging effects by altering the expression of miRs; and proposes that a similar mechanism of damage by these biochemical events may be found in hearing loss; and (c) present data to show that antioxidants protect mammalian cells against oxidative by altering the expression of miRs. A similar role of antioxidants in protecting against hearing disorders is put forward. New studies are proposed to fill the gaps in the areas listed above.

## Introduction

MicroRNAs (miRs) are evolutionarily conserved small non-coding single-stranded RNAs of approximately 22 nucleotides in length, and are present in all living organisms including humans (Lee et al., 1993; Wightman et al., 1993; Macfarlane and Murphy, 2010; Londin et al., 2015). The biogenesis of miRs is very complex and involves multiple biochemical steps. The majority of miRs are transcribed by RNA polymerase II (Pol II), while some are transcribed by RNA polymerase III (Pol III) from the non-coding region of the DNA to produce primary miRs (pri-miRs). Pri-miRs undergo a nuclear cleavage by ribonuclease III Drosa to generate precursor-miRs (pre-miRs) that migrate to the cytoplasm where they are further cleaved by ribonuclease III Dicer to ultimately form mature single-stranded miRs with the help of another protein argonaute (Ago; Hutvágner et al., 2001; Lee et al., 2003; Denli et al., 2004; Macfarlane and Murphy, 2010). Each miR binds to its complimentary sequences in the 3′-untranslated region (3′-UTR) of the mRNA, promotes degradation of the mRNA transcript, and prevents translation of the message of protein. In this manner, miRs regulate the translation of pro-apoptotic or anti-apoptotic proteins from their respective mRNAs, depending upon whether they receive damaging or protective signal.

miRs are expressed in the normal inner ear cells, and play an essential role in their development, differentiation and survival (Friedman et al., 2009; Ushakov et al., 2013). In mouse embryonic inner ears, considerable expression of miR-376a-3p, miR-376-b-3p, and miR-376-c-3p that regulate the levels of phosphoribosyl pyrophosphate synthetase 1 (PRPS 1) were found (Yan et al., 2012). The PRPS 1 protein is important for preserving hair cell function. Mutations in the gene coding for this protein are associated with a spectrum of non-syndromic and syndromic forms of hearing loss. Using inner hair cell line (HE1-OC1 cells), miR-96 and miR183 was found to regulate the levels of chloride intracellular channel 5 (CLIC5) protein. Overexpression of these miRs reduced the levels of CLIC5 (Gu et al., 2013). Expression of 157 miRs in the inner ear sensory epithelial cells, and 53 miRs were differently expressed in cochlear and vestibular cells (Elkan-Miller et al., 2011). Among these, miR135b regulates the levels of PSIP1-p75 that had multiple cellular functions including DNA repair (Pradeepa et al., 2014) and attenuates oxidative stress (Basu et al., 2012). Using embryonic inner ear cell line (UB/OC-1), it was shown that overexpression of miR-210 support differentiation from epithelial cells to sensory hair cells (Riccardi et al., 2016). There were 455 miRs common to both cochlear and vestibular sensory epithelial cells, with 30 miRs unique to the cochlea, and 44 miRs unique to the vestibule. Among these, miR675-5p with its target protein Arhgap12, a GTPase activating protein has been identified (Rudnicki et al., 2014). MiR-194 is expressed in spiral ganglia neurons of mouse inner ear where it may play a role in differentiation of neurons (Wang X. R. et al., 2010). The family of miR-183 consisting of mir-96, miR-182, and miR-183 was strongly expressed in the inner ear hair cells where they play a role in differentiation of primary sensory cells (Li et al., 2010; Zhang et al., 2015). MiR-124 regulated the fate of cells in the developing organ of Corti that contains sensory hair cells and supporting cells (Huyghe et al., 2015).

Since miRs are prominently expressed in the inner ear, it is likely that alterations in their expression may be involved in hearing disorders induced by diverse groups of ototoxic agents, such as chronic and intense noise, vibrations, gentamicin, ionizing radiation, cisplatin, large doses of aspirin, bacterial and viral infection, gene mutations and advanced age. Indeed, changes in the expression of miRs occur in hearing disorders but not understood what signals cause these changes in the expression of miRs that play a role in the pathogenesis of hearing loss. In neurodegenerative disease such as Alzheimer’s disease (AD), increased oxidative stress and products of chronic inflammation such as pro-inflammatory cytokines act as one of the signals that mediate the damaging effects of these biochemical events on the non-auditory neurons (Prasad, 2017). Since increased oxidative stress (Clerici et al., 1995; Van Campen et al., 2002; Henderson et al., 2006; Neri et al., 2006; Vlajkovic et al., 2013; Turcot et al., 2015) and inflammation (Aminpour et al., 2005; Masuda et al., 2006, 2012; Kim et al., 2008; Sziklai et al., 2009; Yamamoto et al., 2009; Verschuur et al., 2014) play a central role in the pathogenesis of hearing defects induced by the ototoxic agents, it is likely that reactive oxygen species (ROS) and pro-inflammatory cytokines may mediate their damaging effects on the hair cells by altering the expression of miRs. This possibility is supported by the fact that the damaging effects of ROS and pro-inflammatory cytokines on the non-auditory neurons are mediated by altering the expression of miRs (Prasad, 2017).

In the non-auditory cells, antioxidants in addition to donating an electron to the molecules with an unpaired electron, they may also mediate their protective effects against oxidative damage by altering the expression of miRs (Prasad, 2017). Since antioxidants reduced the risk of developing hearing defects presumably by decreasing oxidative stress (Sato, 1988; Hou et al., 2003; Kalkanis et al., 2004; Angeli et al., 2005; Husain et al., 2005; McFadden et al., 2005; Kopke et al., 2007; Savastano et al., 2007; Haase et al., 2011; Kapoor et al., 2011; Kang et al., 2013; Seidman et al., 2013; Ojano-Dirain et al., 2014; Kaya et al., 2015), it is likely that protective mechanisms of antioxidants in the auditory cells may be mediated via altering the expression of miRs.

This review article describes studies on alterations in the expression of miRs in the pathogenesis of hearing disorders. It offers evidence from the studies on the non-auditory cells (neurons and glia cells) to support the idea that oxidative stress and pro-inflammatory cytokines may mediate their damaging effects by altering the expression of miRs and proposes studies to demonstrate a similar role of these biochemical events in the auditory cells. This review article also discusses data from the non-auditory cells to show that the protective effects of antioxidants against oxidative damage are mediated in part by altering the expression of miRs. Parallel investigations are needed to look for a similar role of antioxidants in protecting against hearing disorders.

## Alterations in miRs Expression in Hearing Disorders

1. Age-related hearing loss is caused by the cochlear degeneration. Overexpression of miR-29b induced degeneration of cochlear hair cells by decreasing the levels of its target proteins. These key proteins are: (a) silent mating type information regulation 2 homolog 1 (SIRT1), a NAD+ dependent protein deacetylase, which down regulates inflammatory processes; and (b) proliferator-activated receptor-gamma coactivator 1α (PGC-1α). PGC-1α is a stimulator of mitochondrial biogenesis and a regulator of energy metabolism, whose inhibition can lead to impaired mitochondrial function and cochlear hair cell apoptosis in mice (Xue et al., 2016). This study was confirmed by the opposite experiment where inhibition of miR-29b increased the levels of SIRT1 and PGC-1α, and deceased apoptosis of cochlear hair cells (HEI-OC1 inner ear cell line).
2. The expression of Dicer, a ribonuclease III that cleaves pre-miR to form mature miR in the cytoplasm was downregulated in the whole blood of patients with idiopathic sudden sensorineural hearing loss (SSNHL; Kim et al., 2015). Thus, the expression of miRs may also be reduced in this form of hearing loss. However, the expression of Drosha was not altered in the whole blood of these patients suggesting that the processing of miRs at the nuclear level was not affected. This suggests that in SSNHL, alterations in controlling the expression of miRs occur at the level of cytoplasm and not at the nucleus.
3. The expression of miR-34a which causes apoptosis, increased in the cochlea of mice as function of aging, whereas the levels of SIRT1 decreased in these animals. Overexpression of miR-34a also inhibited SIRT1in the inner hair cell line (HE1-OCI). Resveratrol, an activator of SIRT1, decreased miR-34a overexpression, protected hair cells, and reduced hearing loss in mice (Xiong et al., 2015).
4. Degeneration of the organ of Corti, the auditory hair cells that transduces mechanical stimuli to electrical signal in the inner ear, is the major cause of age-related hearing loss. The expression of miR-29 family and miR-34 family that regulate pro-apoptotic pathways was upregulated with aging. However, members of miR-181 and miR-183 responsible for proliferation and differentiation were downregulated during age-related hearing loss (Zhang et al., 2013).
5. MiR-431, which targets the protein Eya4 is highly expressed in the spinal ganglion neurons (SGNs) of the cochlea of newborn mice, and decreases during further development. Inhibition of the cochlear Eya4 protein in miR-431 overexpressing mice led to apoptosis of SGNs and caused hearing loss (Fan et al., 2016).

## Mutation in miR Induces NSHL

Point mutation in miR-96 has been associated with the progressive hearing loss in hereditary nonsyndromic hearing loss (NSHL) both in humans and mice (Friedman and Avraham, 2009; Kuhn et al., 2011). Treatment of mice with N-ethyl-N-nitrosurea (ENU) resulted in mutation in miR-96 that caused hearing loss associated with the damage to the hair cell function and differentiation (Lewis et al., 2009). The mouse model carrying mutated miR-96 is referred to as diminuendo and is considered a good model to study mechanisms of hearing loss. Mutation in miR-96 was also found in two Spanish families with autosomal dominant NSH (Soldà et al., 2012).

## Changes in The Expression of miRs in Noise-Induced Hearing Loss

1. Exposure to intense noise that caused damage to the cochlear hair cells, led to simultaneous decrease of miR-183 levels and corresponding enhancement of its target protein Taok1 (Tao kinase1, a serine/threonine-proten kinase1). This observation was confirmed in the cochlear organotypic culture where inhibition of miR-183 expression resulted in enhanced levels of Taok1 together with apoptosis of hair cells (Patel et al., 2013). Exposure to noise downregulated the expression of miR-176 leading to increased Taok1 levels and apoptosis of the cochlear sensory epithelial cells (Patel et al., 2013).
2. The expression of three miRs: miR-183, miR-96 and miR-182 was decreased 28 days after exposure to noise. This was associated with reduced number of outer hair cells many of which were damaged (Zhang Z. et al., 2014).
3. In male textile workers with noise-induced hearing loss, the plasma levels of miR-16-5p, miR-24-3p, miR-185-5p and miR-451a were upregulated in comparison to those who were exposed to noise exposure but had not developed hearing loss, whereas the plasma levels of miR-24-3p and miR-185-5p and miR-451a were downregulated in individuals exposed to noise in relative to those not exposed (Ding et al., 2016). The protein targets for these miRs were not identified. Plasma levels of these miRs could be of diagnostic value of noise exposure-induced hearing disorders as well as to noise exposure. Further studies are needed to confirm these results.

## Changes in The Expression of miRs in Kanamycin-Induced Hearing Disorders

Treatment of mice with kanamycin increased the expression of miR-34a and miR-34c, and induced apoptosis in the cochlear hair cells, including stria vascularis cells, supporting cells and SGNs (Yu et al., 2010). These effects of kanamycin were associated with increased levels of calpain.

## Alterations in The Expression of miRs in Damaged Auditory Nervous System

Progressive degeneration of SGNs caused sensorineural hearing loss (SNHL). Overexpression of miR-204 suppressed the viability of SGNs by reducing the levels of its target transmembrane protease, serine-3 (TMPRSS3; Li et al., 2014). Therefore, reducing the expression of miR-204 may prevent the development of SNHL. Mutation in the TMPRSS3 gene also caused non-syndromic autosomal recessive deafness with bilateral hearing loss *in utero* or in immature mice. This disease is characterized by degeneration of the organ of Corti and cochlear hair cell loss. Reducing the expression of miR-204 may also help to decrease the development of SNHL and may constitute a therapeutic approach to non-syndromic autosomal recessive deafness.

It is not established, whether changes in the expression of specific miRs are due to alterations in the rate of their transcription, processing by Drosha in the nucleus and Dicer in the cytoplasm or their stability.

Additional studies are needed to explore the effects of different ototoxic agents on the expression of miRs and their respective target proteins in the hair cells and SGNs in culture as well as in animals. In addition, the blood levels of miRs in patients with established hearing loss, and those who have been exposed to a ototoxic agent, but have not developed hearing defects, would also be useful and could readily be investigated.

Table 1 summarizes the alterations in expression of miRs in hearing loss induced by diverse agents and adverse health conditions.

**Table 1:** Upregulated and downregulated microRNAs (miRs) in induced-hearing loss.

Inducing agents	Upregulated microRNAs	Target proteins
Age	miR-29b, miR-34a	SIRT1, PGC-1α
	miR-29 and miR-34 families	
	miR-431	Eya4
Noise	**miR-16-5p, miR-24-3p, miR-185p5	Not identified
	miR-451a	
SNHL	miR-204	TMPRSS3
	**Downregulated microRNAs**	
NSHL	mutated miR-96	Not identified
SSNHL	Dicer	
Noise	miR181, miR-176	Toak1
	*miR-96, mir-182, miR-183	Not identified
	miR-24-3p, miR-185-5p, miR-451a	

SIRT1, Silent mating type information regulation 2 homolog 1; PGC-1α, Proliferator-activated receptor-gamma coactivator1α; Eya4, Eye absent homolog protein4; TMPRSS3, Transmembrane protease, serine-3; Taok1, Tao kinase1 (serine/theorine-protein kinase1); SNHL, Sensorineural hearing loss; SSNHL, Sudden sensorineural hearing loss; NSHL, Nonsyndromic hearing loss. **Levels of noise exposure leading to hearing loss, *Levels of noise exposure not causing hearing loss.

## Oxidative Stress Regulates The Expression of miRs

As mentioned earlier in this manuscript, increased oxidative stress is important in the development of hearing defects and alterations in the expression of miRs occur in this disease. Therefore, it is likely that damaging effects of oxidative stress may be mediated by such alterations in hearing disorders. This is substantiated by the studies on auditory and non-auditory cells. These studies are briefly described here.

### Auditory Cells

Although increased oxidative stress is involved in the pathogenesis of hearing disorders, only a few studies are available on the effects of this biochemical event on changes in the expression of miRs in the cochlear hair cells.

1. Increased oxidative stress induced by tert-butylhydroperoxide (t-BHP) enhanced the expression of 24 miRs. Among these, six miRs miR-1934, miR-411, miR-717, miR-503, miR-467e and miR-699o that regulate apoptosis and proliferation of the cochlear hair cells were strongly expressed (Wang et al., 2016). ROS generated by the treatment with t-BHO increased the expression of 35 miRs and decreased the expression of 40 miRs, and inhibited the proliferation of hair cells (HE1-C1; Wang Z. et al., 2010).
2. ROS generated by exposure to ionizing radiation enhanced the expression of miR-207 in the hair cell line (HE1-OC1). This microRNA increased radiation-induced apoptosis and DNA damage by inhibiting its target protein AKt3. This was further supported by the fact that inhibiting the levels of AKt3 mimicked the effects of miR-207 (Tan et al., 2014).
3. In diabetic mice with high a high level of internal oxidative stress, the expression of miR34a was elevated and this inhibited the levels of SIRT1, increased HIF-1alpha, and promoted apoptosis in the hair cells (HE1-OC1). This finding indicated that downregulation of miR-34a may help in diabetic-related hearing loss (Lin et al., 2017).

### Non-Auditory Cells (Neurons and Non-Neuronal Cells)

In the non auditory cells (neuronal and non-neuronal cells) oxidative stress mediates its damaging effects by altering the expression of miRs (Prasad, 2017).

1. Treatment of human neurons in culture with hydrogen peroxide (H_2_O_2_), upregulated the levels of miR-153 which decreased Nrf2 levels by binding to its 3′-UTR mRNA leading to increased oxidative damage. This was supported by the fact that a mutation in miR-153, anti-miR-153 compound or Nrf2 cDNA devoid of 3′-UTR site protected neurons from H_2_O_2_-induced oxidative damage by activating Nrf2 (Narasimhan et al., 2014 #141). Reduction in the levels of Nrf2 would lead to reduction in the amounts of cytoprotective enzymes including antioxidant enzymes and phase-2-detoxifying enzymes leading to increased oxidative stress.
2. Treatment of rat spinal cord neurons with H_2_O_2_ upregulated the expression of miR-146 a, miR-21 and miR-150 and this resulted in apoptosis of neurons. Silencing of only miR-21 expression diminished H_2_O_2_-induced cell death by decreasing oxidative stress (Jiao et al., 2015). Conversely, increased expression of miR-21 reduced the levels of Nrf2 by binding to its 3′-UTR mRNA site.
3. Hyperoxia increased the expression levels of miR-185 and caused cell death in human lung epithelial cells in culture (Zhang et al., 2016). This upregulation of miR-185 inhibited histone deacetylase-4 and led to DNA damage and apoptosis. MC1586, an inhibitor of histone deacetylases, increased the expression of miR-185, and this also caused cell death (Zhang et al., 2016).
4. Oxidative damage induces premature senescence in an aging animal model. Oxidative stress increased the expression of miR-24 and reduced the levels of DNA topoisomerase-1 (TOP1) levels. TOP1 is an enzyme regulating DNA topology, and reduction of it’s concentration led to senescence of fibroblasts (Bu et al., 2016). TOP1 is thus likely to be the target protein for miR24.

These reports indicate that ROS may be one of the signals that regulates the expression of key miRs that, by decreasing the action of their target proteins namely Nrf2, TOP1 and histone deacetylase, leads to degeneration of neurons.

Additional studies should be performed on the effects of ROS donors on the expression of miRs in cultured hair cells and SGNs, and in animals following exposure to ROS donors. In addition, the levels of markers of oxidative damage and expression of miRs could be looked for in the blood of patients with established hearing defects as well as in individuals exposed to ototoxic agents, but have not yet developed deafness.

Table 2 summarizes the effects of ROS on the expression of miRs.

**Table 2:** Reactive oxygen species (ROS) and pro-inflammatory cytokine alter the expression of miRs in neurons.

ROS-induced upregulation of microRNAs	Target proteins
MiR-21, miR24, miR-146a, miR-150, miR-153 and miR-185,	Nrf2, TOP 1 and histone deacetylase
**Pro-inflammatory cytokine-induced upregulation of microRNAs**	
MiR-7, mirR-9, miR-34a, miR-125b, miR-146a, miR-155	Not identified

*Nrf2, Nuclear transcriptional factor-2. TOP1, DNA topoisomerase 1. This table was reproduced from a previous publication (Prasad, 2017)*.

## Pro-Inflammatory Cytokines Upregulate Expression of miRs

As mentioned earlier in this manuscript, inflammation is involved in the pathogenesis of hearing defects and alterations in the expression of miRs occur in this disease. Therefore, it is likely that damaging effects of inflammation may be mediated by such alterations in hearing loss. This possibility is supported by the investigations on non-auditory cells (neurons and astroglia) showing that pro-inflammatory cytokines bring about their damaging effects in part, by altering the expression of miRs (Prasad, 2017). These studies are briefly described here.

1. ROS and pro-inflammatory NF-κB induced by a combination of iron and aluminum sulfate) upregulated the expression of miR-125b and miR-146a in cultured human astroglial cells (Pogue et al., 2011). Phenylbutyl nitrone, an antioxidant; and curcumin, an inhibitor of NF-κB, both prevented this upregulation (Pogue et al., 2011). Treatment of human astroglial cells in culture with IL-1β, a pro-inflammatory cytokine elevated the expression of miR-146a causing cell death. These findings show that pro-inflammatory cytokines act as one of the signals that upregulate miRs.
2. The pro-inflammatory transcriptional factor NF-κB upregulated the levels of miR-7, miR-9, miR-34a, miR-125b, miR-146a and miR-155 in cultured human neuronal-glial cells (Zhao et al., 2015). Treatment of human primary neurons in culture with TNF-α increased the expression of miR-146a and miR-155 (Lukiw et al., 2012).

These studies show that pro-inflammatory cytokines mediate their degenerative effects on neurons and glia cells at least in part by upregulating the expression of miRs. Studies similar to those proposed under the section of oxidative stress should be performed on the auditory cells exposed to pro-inflammatory cytokines.

Table 2 summarizes the effects of pro-inflammatory cytokines on the expression of miRs.

## Antioxidants Alter The Expression of miRs

Antioxidants protect hair cells against oxidative damage and prevent hearing loss (Prasad, 2011). Studies on non-auditory cells revealed that the protective action of antioxidants against oxidative damage was in part mediated by altering the expression of miRs (Prasad, 2017). Thus, it is likely that antioxidants may also protect the inner ear cells against inflammatory damage by altering the expression of miRs. Investigations on the effects of antioxidants on the expression of miRs in non-auditory cells are described here.

### Resveratrol-Induced Increase in the Expression of microRNAs

Treatment with resveratrol upregulated the expression of miR-328, and in consequence inhibited the production of its target protein metalloproteinase-2 (MMP-2) in osteosarcoma cells (Yang et al., 2015). Such treatment also enhanced the expression levels of miR-137, with a corresponding inhibition of its target protein, histone methyltransferease enhancer of Zest 2 polycomb repressive complex 2 subunit (EZH2) in neuroblastoma cells (Ren et al., 2015). Resveratrol treatment of cultured human colon cancer cells increased the expression of miR-663, which decreased the levels of its target proteins, program cell death protein4 (PDCD4), phosphatase and tensin homolog (PTEN) and transforming growth factor (TGF; Tili et al., 2010). In a transformed human bronchial epithelial cell line, resveratrol treatment also upregulated miR-622 resulting in inhibition of its target protein K-ras (Han et al., 2012). In the peripheral blood mononuclear cells of hypertensive male patients with type 2 diabetes, daily oral supplementation with grape seed extract rich in resveratrol for a year increased the expression of miR-21, miR-181b, miR-663, miR-30c2, miR-155 and miR-34a and thereby reduced the levels of their target pro-inflammatory cytokines C-C motif chemokine ligand 3 (CCl3), IL-1β and TNF-α (Tomé-Carneiro et al., 2013).

### Resveratrol-Induced Decrease in the Expression of microRNAs

Resveratrol treatment reduced the expression levels of miR-134 and miR-124. Since cyclic-AMP response binding protein (CREB), a nuclear transcriptional factor, is one of their target proteins, their decreased expression led to production of increased levels CREB leading to enhanced synthesis of BDNF (Zhao et al., 2013). Resveratrol treatment decreased the expression of miR-21 in several cancers cells in culture (Sheth et al., 2012; Li et al., 2013; Liu et al., 2013; Zhou et al., 2014), and miR-33a and miR-122 in isolated hepatic cells (Baselga-Escudero et al., 2014). Treatment with grape seed extract deceased the expression of miR-27a which increased the levels of its target protein Forkhead box protein O1 (FOXO1; Ma et al., 2015). This protein can reduce stress response.

### Isoflavone-Induced Increase in the Expression of microRNAs

Isoflavone treatment increased the expression of miR-200b, miR-200c, miR-let-7b, miR-let-7c, miR-let-7d, and miR-let-7e resulting in reduced levels of their target proteins zinc finger E-box-binding homeobox 1 (ZEB1) and vimentin in pancreatic cancer cells (Li et al., 2009). In cultured breast cancer cells, treatment with the natural compounds, such as enoxolone (or glycyrrhetinic acid), a component of licorice with anti-viral, anti-bacterial and antifungal activities, and magnolol, a component of the bark of *Magnolia officinalis*, upregulated the expression levels of miR-200c which inhibited its target protein ZEB1 (Hagiwara et al., 2015). These changes in the expression of miRs were associated with reduced growth of cancer cells.

### Genistein-Induced Decrease in the Expression of microRNAs

Treatment of cultured pancreatic cancer cells with genistein downregulated the expression of miR-223 and miR-34a which increased the levels of their target proteins F-box and WD repeat domain-containing 7 (Fbw7) and Notch-1 and reduced growth (Xia et al., 2012; Ma et al., 2013).

### Quercetin-Induced Increase in the Expression of microRNAs

The growth of breast cancer cells in culture was inhibited after treatment with quercetin. This agent also enhanced the expression of miR-146a (Tao et al., 2015). Quercetin upregulated the expression of hepatic miR-122 and miR-125b and this was associated with decreased levels of inflammatory genes (Boesch-Saadatmandi et al., 2012).

### Curcumin-Induced Decrease in the Expression of microRNAs

In a human primary culture of neuronal-glia cells, curcumin treatment prevented ROS and NF-κB-induced upregulation of miR-125b and miR-146a (Pogue et al., 2011). The pro-inflammatory cytokine IL-1β increased the expression of miR-146a; however, curcumin treatment prevented IL-1β-induced upregulation of miR-146a in human primary culture of neuronal-glial cells (Li et al., 2011). This implies a reciprocal regulatory relationship between miRs and their target proteins. Increased expression of miR-146a was associated with enhanced senile plaque density and synaptic pathology in transgenic mouse models of AD (Tg2576 and 5XFAD). Curcumin suppressed the expression of miR-21 and elevated its target protein PTEN in human non-small cell lung carcinoma (Zhang W. et al., 2014).

### Curcumin-Induced Increase in the Expression of microRNAs

Curcumin increased the expression of miR-22 (Sun et al., 2008) in human pancreatic cancer cells. Curcumin-induced upregulation of miR-22, and thereby reduced the level of its target protein transcriptional factor-1 (SP-1) by binding to its 3′-UTR mRNA. Silencing the expression of miR-22 enhanced the levels of SP-1. Treatment of cultured bladder cancer cells with curcumin upregulated the expression of miR-203, which reduced the levels of its target proteins protein kinase B (Akt2) and src tyrosine protein kinase (Saini et al., 2011). In breast cancer cells, treatment with curcumin upregulated the expression of miR-7, which decreased the concentration of its target protein SET8 histone lysine methyltransferase. Curcumin increased the expression of miR-181b and reduced the levels of its target protein chemokine (C-X-C Motif) ligand-1 (CXCL-1; Kronski et al., 2014). Alterations in the expression of miRs were associated with growth inhibition.

### Coenzyme Q10- and N-Acetylcysteine-Induced Decrease in the Expression of microRNAs

In primary culture of umbilical vein endothelial cells, treatment with coenzyme Q10 reduced lipopolysaccharide (LPS)-induced elevation of the expression of miR-146a and corresponding reduction of its target protein IL-1receptor associated kinase-1 (ILRAL-1; Olivieri et al., 2013).

Increased oxidative stress induced by exposure to diesel fuel upregulated the expression of miR-21, miR-30e, miR-215 and miR-144 in the blood of patients with mild asthma. The upregulation of miR-144 resulted in reduction of its target protein Nrf2 and this then caused decreased levels of glutamate cysteine ligase catalytic subunit (GCLC) and NAD(P) H: quinone oxidoreduxtase-1 (NQO1). Treatment with N-acetylcysteine (NAC) attenuated this diesel fuel-induced elevation of oxidative stress and the expression of miR-144 and miR-21 (Yamamoto et al., 2013).

The studies discussed above suggest that different antioxidants affect the expression and type of miRs in a variety ways. Some antioxidants (resveratrol and curcumin) can both increase and decrease the expression of miRs, others (isoflavone and quercetin) are only reported to enhance them, while others (genistein and NAC) may only decrease them. It is interesting to note that resveratrol treatment upregulates and downregulates the expression levels of miR-21, whereas curcumin and NAC only downregulate the expression of miR-21. Upregulation of miR-21 appears to reduce the levels of pro-inflammatory cytokines, whereas its downregulation reduces the intensity of oxidative stress by enhancing Nrf2 level. The same microRNA appears to target the mRNAs for several proteins thus facilitating a decrease in both oxidative stress and inflammation.

The upregulation and downregulation of miR-21 by resveratrol involve different target proteins but the overall effect is the reduction of oxidative stress and inflammatory processes. Similarly, miR146a was upregulated by the quercetin treatment, while it was down regulated by the treatment with curcumin and coenzyme Q10. However differing target proteins lead to the amelioration of oxidant and inflammatory event in either case.

New investigations on the effect of antioxidants on changes in the expression of miRs in cultured hair cells and SGNs and in animals following treatment with ototoxic agents should be performed.

Table 3 summarizes data on the effects of antioxidants on changes in the expression of miRs in non-auditory cells.

**Table 3:** Effects of antioxidants compounds on the expression of miRs.

Antioxidants	Upregulation	Downregulation
Resveratrol	miR-21, miR-30c2, miR-34a, miR-137, miR-155, miR-181b, miR-328, miR-622, miR-663	miR-21, miR-27a, miR-33a, miR-122, miR-124, miR-134
Curcumin	miR-7, miR-22, miR-181b, miR-203	miR-21, miR-125b miR-146a
Isoflavone	miR-let-7b, miR-let-7c, miR-let-7d miR-let-7e, miR-200b, miR-200c	None
Genistein	None	miR-34a, miR-223
Quercetin	miR-122, miR-125b, miR-146a	None
Coenzyme Q10	None	miR-146a
N-acetylcysteine	None	miR-21, miR-144

*It is interesting to note that resveratrol treatment upregulated and downregulated the expression levels of miR-21, whereas curcumin and N-acetylcysteine downregulated the expression levels of miR-21. Upregulated and downregulated miR-21 used different target proteins to reduce oxidative stress and inflammation. Similarly, miR-146a was upregulated by the quercetin treatment, whereas it was downregulated by the treatment with curcumin and coenzyme Q10. This table was reproduced from a previous publication (Prasad, 2017)*.

## Conclusion

Increased oxidative stress and chronic inflammation play an important role in the initiation and progression of hearing loss induced by diverse ototoxic agents or adverse health conditions. Some studies show that the expression of miRs is altered in hearing loss. Although increased oxidative damage and inflammation are involved in the pathogenesis of hearing disorders, the relation of these biochemical events to the expression of miRs on auditory cells remains unclear. However, in case of the non-auditory cells (neurons and glia cells), ROS and pro-inflammatory cytokines mediate their degenerative effects to some extent by changing the expression of miRs in a way that allows increased production of pro-apoptotic proteins. Therefore, it is likely that a parallel mechanism of degeneration of the auditory system by these processes may be found in hearing disorders. While antioxidants protect against oxidative damage in hearing disorders, it is unknown whether they provide such protection by altering the expression of miRs. The fact that antioxidants protect the non-auditory cells against oxidative damage by altering the expression of miRs suggests that a similar mechanism of protection by antioxidants may exist in hearing disorders. New studies are suggested to fill the gaps that exist on the understanding the role of miRs in the pathogenesis of hearing disorders.

## Author Contributions

SCB has contributed to the writing and discussion of the contents of the manuscript. KNP is responsible for the concept, research, and writing the manuscript.

## Conflict of Interest Statement

The author KNP is the Chief Scientific Officer of Engage Global that distributes nutritional products. The other author declares that the research was conducted in the absence of any commercial or financial relationships that could be construed as a potential conflict of interest.
